# Contralateral Lower Neck Sparing Radiotherapy in Stage N1 Nasopharyngeal Carcinoma: Long-Term Survival Outcomes and Late Toxicities

**DOI:** 10.3389/fonc.2021.628919

**Published:** 2021-02-25

**Authors:** Zhuang Sun, Jingyun Wang, Runda Huang, Xiaohui Wang, Chunyan Chen, Meiling Deng, Chong Zhao, Hanyu Wang, Fei Han

**Affiliations:** ^1^ Department of Radiation Oncology, Sun Yat-sen University Cancer Center, Guangzhou, China; ^2^ State Key Laboratory of Oncology in South China, Guangzhou, China; ^3^ Collaborative Innovation Center for Cancer Medicine, Guangzhou, China; ^4^ Guangdong Key Laboratory of Nasopharyngeal Carcinoma Diagnosis and Therapy, Guangzhou, China; ^5^ Department of Nasopharyngeal Carcinoma, Sun Yat-sen University Cancer Center, Guangzhou, China

**Keywords:** nasopharyngeal carcinoma, stage N1, contralateral lower neck sparing radiotherapy, late toxicities, long-term quality of life

## Abstract

**Purpose:**

To explore the feasibility of contralateral lower neck sparing radiotherapy for patients with stage N1 nasopharyngeal carcinoma (NPC) by analyzing long-term survival outcomes and late toxicities.

**Methods:**

Data of patients with stage N1 NPC who were treated with contralateral lower neck sparing radiotherapy between January 2013 and December 2015 were analyzed. These patients were all staged by magnetic resonance imaging (MRI), and all received irradiation to the upper neck (levels II, III, and Va) bilaterally along with ipsilateral levels IV and Vb, without irradiation of the contralateral lower neck. Treatment outcomes, regional failure patterns, and late toxicities were examined.

**Results:**

A total of 275 eligible patients with stage N1 NPC were included in the present study. The median follow-up period was 62 months (range, 3–93 months). The 5-year overall survival (OS), distant metastasis-free survival (DMFS), local recurrence-free survival (LRFS), regional recurrence-free survival (RRFS), locoregional recurrence-free survival (LRRFS), and progression-free survival (PFS) rates were 90.5, 91.3, 94.7, 95.3, 91.2, and 81.7%, respectively. A total of 13 patients (4.7%) developed regional recurrence, all of which occurred in the field and not out of the field. Among 254 patients with available data on late toxicities, the most common late toxicity was xerostomia. No late injuries occurred in the carotid arteries, brachial plexus, or spinal cord. In addition to one case (0.4%) of neck fibrosis and three cases (1.2%) of hearing loss, there were no other grade 3–4 late toxicities observed.

**Conclusions:**

Contralateral lower neck sparing radiotherapy would be safe and feasible for patients with stage N1 NPC, with the potential to improve the long-term quality of life of patients.

## Introduction

Nasopharyngeal carcinoma (NPC) is a type of epithelial head and neck tumor with definite geographical distribution characteristics and is especially prevalent in East and Southeast Asia (1). For newly diagnosed non-metastatic NPC, radiotherapy is the standard treatment because of its high radiosensitivity. Given the relatively high incidence of cervical lymph node metastasis in NPC (2, 3), in many research protocols, irradiation of the entire bilateral cervical lymphatic drainage area is thought to be warranted irrespective of the lymph node status (4–7). However, extensive neck irradiation may lead to severe late toxicities such as neck subcutaneous fibrosis, hypothyroidism, and carotid stenosis, thus adversely influencing the quality of life of long-term survivors (8–12). Therefore, it is essential to investigate whether omitting the irradiation of certain neck areas would be feasible.

Many studies (13–17) have focused on the efficacy of prophylactic upper neck radiotherapy in patients with stage N0 NPC or with only retropharyngeal lymph node metastasis. However, studies on whether contralateral lower neck sparing radiotherapy would be safe for patients with stage N1 NPC are still scarce. Our team previously reported a study in which we found that only 1.4% of patients with stage N0–1 NPC experienced out-of-field lymph node recurrence when levels IV and Vb was excluded from the irradiation of node-negative necks (18). Although this study provided some evidence to support the radiotherapy approach of sparing the lower neck, it also had some limitations. First, all patients included were diagnosed and staged using computed tomography (CT). Second, the lower necks (levels IV and Vb) were all treated with conventional radiotherapy rather than intensity-modulated radiotherapy (IMRT). Finally, late toxicities associated with neck irradiation received inadequate attention.

Accordingly, we conducted the present study in which we analyzed the therapeutic outcomes and late sequelae of patients with stage N1 NPC who received IMRT but omitted elective neck irradiation to the contralateral lower neck, in a continuing effort to provide further evidence for the practicability of contralateral lower neck sparing radiotherapy in stage N1 NPC in the IMRT era.

## Materials and Methods

### Patients

All patients included in this study were treated at Sun Yat-sen University Cancer Center between January 2013 and December 2015. Inclusion criteria were as follows: (1) newly diagnosed and pathologically proven NPC; (2) undergoing magnetic resonance imaging (MRI) scans of the nasopharynx and neck at diagnosis; (3) T1–4N1M0 disease according to the 8th edition of the American Joint Committee on Cancer (AJCC) Staging Manual; (4) no other concomitant malignant tumors; (5) receiving contralateral lower neck sparing radiotherapy with IMRT technique, that is, bilateral upper neck (levels II, III, and Va) along with levels IV and Vb on the side with cervical lymph node involvement were irradiated, while the contralateral lower neck was not irradiated; (6) data of the target delineation were available. Exclusion criteria were as follows: (1) stage N1 patients with retropharyngeal lymph node metastasis only; (2) receiving excisional nodal biopsy or neck dissection before radiotherapy. This study was approved by the Clinical Research Ethics Committee of Sun Yat-sen University Cancer Center.

### Pretreatment Evaluations

Pretreatment evaluations were performed for all patients, and they underwent a complete physical examination, routine blood test, biochemical examination, as well as nasopharyngoscopy, MRI scan of the nasopharynx and neck, X-rays or CT scan of the chest, and abdominal ultrasound. Positron emission tomography/computed tomography (PET/CT) was also used when there were clinical indications. All enrolled patients were reclassified under the 8th edition of the AJCC Staging Manual.

The diagnostic criteria for metastatic cervical lymph nodes were as follows: (1) the minimal axial diameter of lymph nodes was ≥11 mm in the jugulodigastric region or ≥10 mm in other neck regions; (2) there was a cluster of three or more borderline lymph nodes; and (3) there was imaging proof of necrosis or extracapsular spread regardless of node size (19). The lateral retropharyngeal lymph nodes were deemed positive only when their minimal axial diameter was ≥5 mm. Any visible median retropharyngeal lymph nodes were considered malignant (20). The classification of neck node levels proposed by the Radiation Therapy Oncology Group (RTOG) was adopted (21).

### Radiotherapy

**Figure 1:**
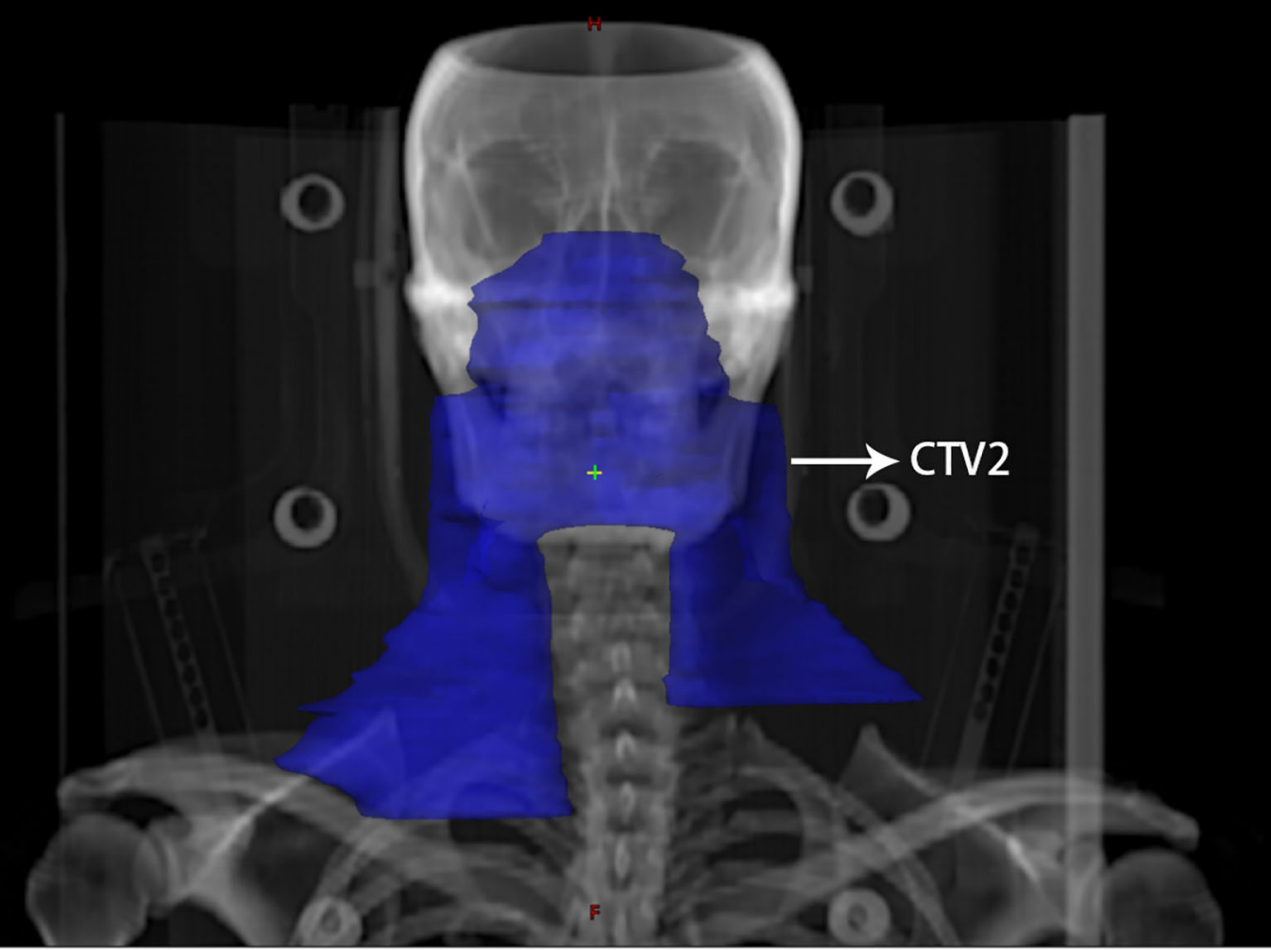
An illustration of contralateral lower neck sparing radiotherapy for a patient with stage N1 nasopharyngeal carcinoma. The blue area indicates the extent of elective neck irradiation, including the bilateral upper neck as well as the ipsilateral lower neck, excluding the lower neck on the contralateral side.

All patients were treated with IMRT once a day for five days a week. The delineation of the target volume was consistent with the International Commission on Radiation Units and Measurements (ICRU) reports 50 and 62 (22). The gross tumor volume (GTV) was determined on the basis of clinical and imaging results, comprising the primary nasopharyngeal tumor (GTVnx) and the lymph nodes involved (GTVnd). The enlarged retropharyngeal lymph node was also included in the GTVnx. The clinical target volume (CTV) included the high-risk clinical target volume (CTV1) and the low-risk clinical target volume (CTV2). CTV1 contained the GTVnx and an added 5–10 mm margin to cover microscopically extended high-risk areas and the entire nasopharynx. CTV2 contained CTV1 as well as an added 5–10 mm margin to cover microscopically extended low-risk areas. In addition, relevant cervical lymph node drainage areas were delineated in CTV2. Of note, for stage N1 patients in this study, bilateral upper neck and ipsilateral levels IV and Vb were included in CTV2, omitting the contralateral lower neck (**Figure 1**).

The prescribed doses were: 68–70 Gy in 30–33 fractions to the planning target volume (PTV) of GTVnx, 64–70 Gy in 30–33 fractions to the PTV of GTVnd, 60 Gy in 30–33 fractions to the PTV of CTV1, and 54 Gy in 30–33 fractions to the PTV of CTV2.

### Chemotherapy

The modes of chemotherapy used were based on the clinical stage of the tumor. Patients with stage II NPC received concurrent chemotherapy. Patients with stages III and IV NPC received induction chemotherapy plus concurrent chemotherapy or concurrent chemotherapy alone.

Induction chemotherapy was administered before radiotherapy, which included the regimens of docetaxel plus cisplatin and fluorouracil (TPF), docetaxel plus cisplatin (TP), cisplatin plus fluorouracil (PF), and gemcitabine plus cisplatin (GP). The regimens of induction chemotherapy were repeated every 3 weeks for a total of 2 or 3 cycles. During radiotherapy, the regimens of concurrent chemotherapy were performed, including single-agent cisplatin (80–100 mg/m^2^ every 3 weeks) and single-agent cisplatin (30–40 mg/m^2^ weekly).

### Follow-up

After treatment, patients were followed up every 3 months for the first 2 years, then every 6 months for the next 3–5 years and annually thereafter. Regular follow-up examinations consisted of physical examination, routine blood test, biochemical examination, nasopharyngoscopy, MRI scan of the nasopharynx and neck, X-rays or CT scan of the chest, and abdominal ultrasound.

At each follow-up, late toxicities were assessed based on the toxicity criteria of RTOG (23) and the Common Terminology Criteria for Adverse Events v4.0.

### Statistical Analysis

The endpoints of this study were as follows: overall survival (OS), distant metastasis-free survival (DMFS), local recurrence-free survival (LRFS), regional recurrence-free survival (RRFS), locoregional recurrence-free survival (LRRFS), and progression-free survival (PFS). All endpoints were counted from the first day of treatment. OS was defined as the interval from the first day of treatment to the last follow-up or death for any cause; DMFS, to the first occurrence of distant metastasis; LRFS, to the first occurrence of local recurrence; RRFS, to the first occurrence of regional recurrence; LRRFS, to the first occurrence of local or regional recurrence; and PFS, to the first disease progression or death for any reason.

Statistical analysis was conducted using IBM SPSS Statistics version 25.0 (IBM Corp, Armonk, NY). The actuarial rates of the endpoints above were calculated using the Kaplan–Meier method and differences between survival rates were compared using the log-rank test. P values <0.05 were considered as statistically significant.

## Results

### Patient Clinical Characteristics

**Figure 2:**
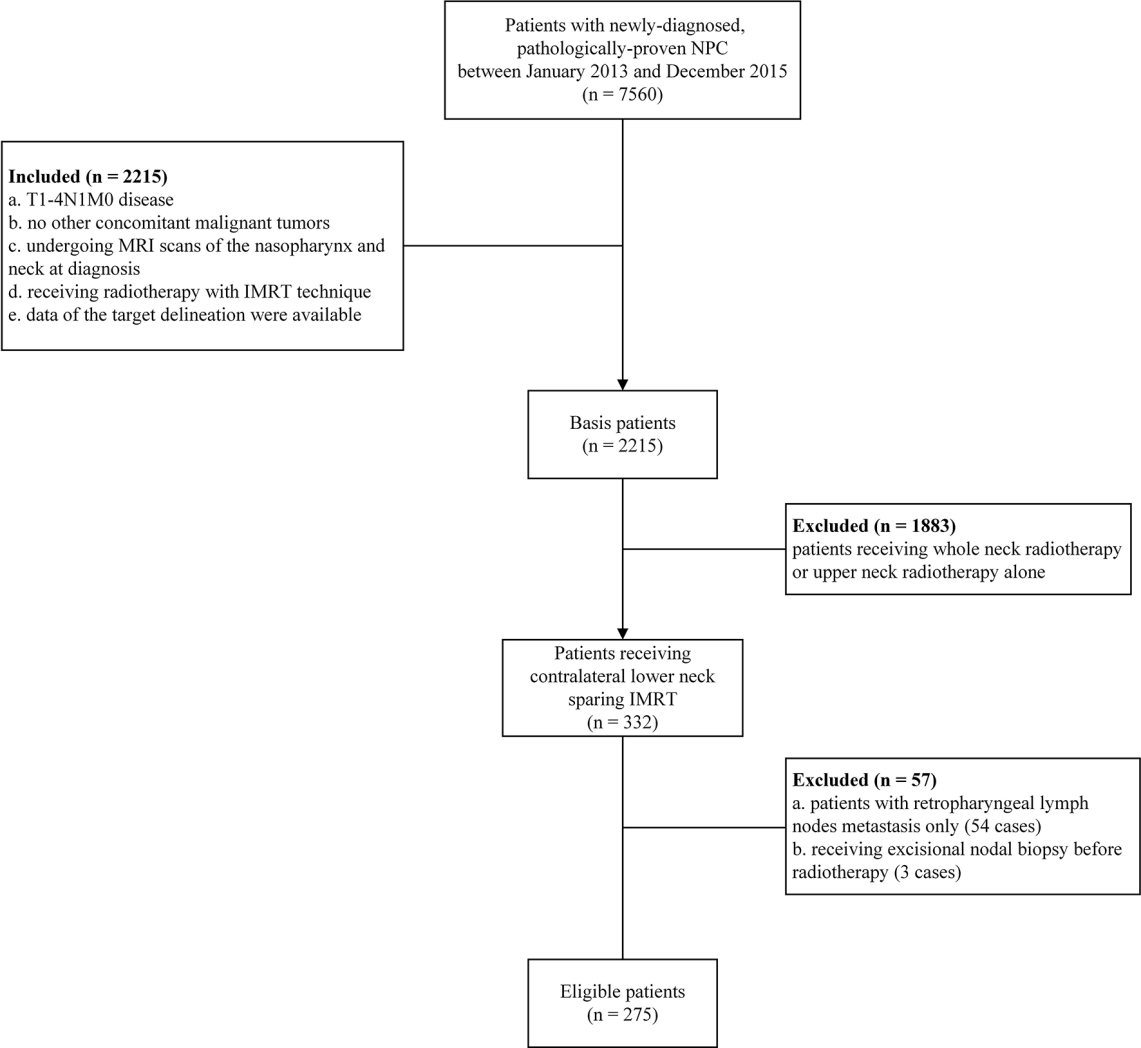
Flowchart of the patients included in the study.

In total, 275 eligible patients with stage N1 disease were included in this study (**Figure 2**). Of the total patients, 182 were male and 93 were female. The median age was 45 years, ranging from 13 to 76 years. Besides, a total of 189 patients (68.7%) in this study exhibited retropharyngeal lymph node metastasis at diagnosis, including 60 patients (21.8%) with contralateral retropharyngeal lymph node metastasis. The detailed clinical characteristics of these patients are summarized in **Table 1**.

**Table 1:** Characteristics of the 275 patients with stage N1 nasopharyngeal carcinoma treated by contralateral lower neck sparing radiotherapy.

Characteristic	No.	%
Sex		
Male	182	66.2
Female	93	33.8
Age(y)		
Median	45	
Range	13–76	
Pathological type		
Nonkeratinizing squamous cell carcinoma	274	99.6
Basaloid squamous cell carcinoma	1	0.4
T stage		
T1	21	7.6
T2	56	20.4
T3	135	49.1
T4	63	22.9
Chemotherapy		
No	29	10.5
Yes	246	89.5
IC alone	17	6.2
CCT alone	135	49.1
IC+CCT	94	34.2
Anti-EGFR targeted therapy^*^		
No	267	97.1
Yes	8	2.9

y, years; IC, induction chemotherapy; CCT, concurrent chemotherapy; EGFR, Epidermal growth factor receptor.

^*^The agents of anti-EGFR targeted therapy comprised cetuximab and nimotuzumab.

Of the patients included in this study, 246 patients (89.5%) received chemotherapy, including 17 patients (6.2%) receiving induction chemotherapy alone, 135 patients (49.1%) receiving concurrent chemotherapy alone, and 94 patients (34.2%) receiving induction chemotherapy plus concurrent chemotherapy. Among patients with stage II NPC, 80.5% (62/77) received chemotherapy. Among patients with stage III or IV NPC, 92.9% (184/198) received chemotherapy.

### Treatment Outcomes

The mean follow-up time was 62 months (range, 3–93 months). Overall, 23 patients (8.4%) developed distant metastases, which was the most common failure pattern. Moreover, 15 cases (5.5%) of local recurrence and 13 cases (4.7%) of regional recurrence were recorded. **Table 2** lists the detailed failure modes. By the last follow-up, a total of 28 patients (10.2%) died, with the majority (23/28, 82.1%) ascribed to NPC.

**Table 2:** Failure patterns of treatment in the 275 patients with stage N1 nasopharyngeal carcinoma treated by contralateral lower neck sparing radiotherapy.

Patterns of failure	No.	%
Local and/or regional recurrence	24	8.7
Local recurrence only	11	4.0
Regional recurrence only	9	3.3
Local and regional recurrence	4	1.5
Distant metastasis	23	8.4
Distant metastasis only	20	7.3
Distant metastasis + Local recurrence	1	0.4
Distant metastasis + Regional recurrence	0	0.0
Distant metastasis + Local recurrence +Regional recurrence	2	0.7

The 5-year OS, DMFS, LRFS, RRFS, LRRFS, and PFS rates were 90.5, 91.3, 94.7, 95.3, 91.2, and 81.7%, respectively. In addition, there were no significant differences between patients without contralateral retropharyngeal lymph node metastasis and those with contralateral retropharyngeal lymph node metastasis in the 5-year RRFS (94.6 *vs*. 98.2%, *P =* 0.25).

### Patterns of Regional Recurrence

Overall, 13 patients (4.7%) experienced regional recurrence. All cases were in-field regional failure, and none of them had out-of-field regional failure. **Table 3** summarizes the patterns of regional recurrence in detail. The sites of regional recurrence were concentrated in levels II and III. No patients experienced regional recurrence in levels IV or V. The median time to regional recurrence was 22 months (range, 14–72 months).

**Table 3:** Patterns of regional recurrence of the 275 patients with stage N1 nasopharyngeal carcinoma treated by contralateral lower neck sparing radiotherapy (n = 13).

No.	Sex	Age (y)	T stage	Initial level involved	Regional recurrence site	Failure pattern	Time to regional recurrence (m)
1	Female	51	T4	Right II	Right II	In-filed	20
2	Female	42	T4	Right II	Bilateral II, Right III	In-filed	18
3	Male	41	T4	Bilateral RLN, Left II	Left II	In-filed	22
4	Male	63	T3	Left RLN, II, III	Left II	In-filed	14
5	Male	41	T3	Left RLN, II, III	Left II	In-filed	14
6	Male	43	T3	Left II, III	Left III	In-filed	49
7	Male	49	T4	Left RLN, II	Left II	In-filed	49
8	Male	44	T1	Right II	Right II	In-filed	21
9	Female	47	T2	Left II	Left II	In-filed	72
10	Female	45	T2	Left RLN, II, III	Left II	In-filed	33
11	Male	53	T4	Left II	Left II	In-filed	22
12	Male	38	T2	Left II, III	Left II	In-filed	14
13	Female	53	T2	Right II	Right II	In-filed	30

y, years; m, months; RLN, retropharyngeal lymph node.

### Late Toxicities

In total, data on late toxicities of 254 patients (92.4%) were available. Most late toxicities were assessed as grade 0 or grade 1, and the most common late toxicity was xerostomia. No late injuries were observed in the carotid arteries, brachial plexus, or spinal cord. Grade 3–4 late toxicities were recorded in one case (1/254, 0.4%) of neck fibrosis and three cases (3/254, 1.2%) of hearing loss. In addition, 70 patients were evaluated for serum thyroid function after IMRT. Of these 70 patients, five cases (5/70, 7.1%) of overt hypothyroidism and 22 cases (22/70, 31.4%) of subclinical hypothyroidism were found.

## Discussion

The entire bilateral neck area has long been recommended for irradiation in patients with NPC regardless of the status of nodal metastasis to achieve adequate regional control (4–7). However, it should be noted that this recommendation is based on clinical experience and the results of a few retrospective studies in the era of conventional radiotherapy (24, 25). Moreover, the lymph nodes of most patients were diagnosed by clinical palpation and traditional CT scan in the past, which might lead to missed diagnosis. Currently, modern imaging techniques such as MRI and PET/CT have been essential in the diagnosis and staging of NPC and they have improved the understanding of lymph node diffusion patterns. A study based on 3,100 patients with NPC who underwent MRI showed that NPC follows an orderly lymphatic spread pattern from higher levels to lower levels. The most frequent sites of lymph node metastases were level II (87.4%) and the retropharyngeal area (75.1%), followed by level III (44.2%), level V (37.1%), and level IV (14.1%) (26). In addition, the meta-analysis of Ho et al. (27) demonstrated that skip metastasis of lymph nodes is relatively rare, with an incidence ranging from 0.2 to 7.9%. More importantly, wide-range irradiation of the whole neck could result in dysfunction in surrounding critical organs and tissues and affect the patient’s long-term quality of life (8–12). Therefore, it is logical to question whether radiotherapy covering the entire neck is necessary.

Recently, an increasing number of studies have focused on how to minimize the irradiation range of the neck and improve the quality of life of long-term patients. Some studies have shown that elective irradiation of the bilateral upper neck alone is feasible for patients with stage N0 NPC (13–15) or with only retropharyngeal lymph node metastasis (16, 17). Furthermore, in one of our previous studies (18), the impact of omitting irradiation to levels IV and Vb in node-negative necks was evaluated. In addition to 128 N0 patients, the study included 84 patients with N1 NPC staged by CT. At a median follow-up time of 59 months, only 0.5% of patients experienced lymph node recurrence at the omitted level Vb, and none had lymph node failure at level IV. Hu et al. (28) investigated the treatment efficacy of 52 patients with stage N1 disease who received irradiation of bilateral upper neck and ipsilateral levels IV and Vb but omitted the contralateral lower neck. With a median follow-up time of 29 months, only one patient had regional failure in the irradiated area (level II), whereas no patient developed out-of-field nodal failure. The reported 3-year OS, LRFS, RRFS, and DMFS rates were 92.2, 94.3, 98, and 94.1%, respectively. Although important evidence for the practicability of sparing radiotherapy of the contralateral lower neck was provided by the two studies above, there are also limitations of the relatively small number of patients enrolled and the short follow-up time.

In this study, the data of 275 patients with stage N1 NPC who received contralateral lower neck sparing radiotherapy were analyzed. The 5-year OS, DMFS, LRFS, RRFS, LRRFS, and PFS rates were 90.5, 91.3, 94.7, 95.3, 91.2 and 81.7%, respectively. Of particular note, only 13 cases (4.7%) of cervical lymph node recurrence occurred in the irradiated field and none developed out-of-field nodal recurrence. Compared with the results of other studies (29, 30), our radiotherapy approach did not have a negative effect on regional control nor declined the long-term survival rates of patients. In addition, we found that the presence of contralateral retropharyngeal lymph node metastasis would not impair the regional control of patients who were treated with contralateral lower neck sparing radiotherapy, since there were no significant differences in the 5-year RRFS rates between patients with contralateral retropharyngeal lymph node metastasis and those without contralateral retropharyngeal lymph node metastasis.

Notedly, the incidence of severe late toxicities associated with neck irradiation, including neck fibrosis, hypothyroidism in patients in this study was relatively low when compared with the data of late toxicities in previous studies using bilateral whole neck irradiation (10, 31–34). For example, the incidence of grade 3 neck fibrosis for patients receiving bilateral whole neck irradiation by IMRT was reported to be 4.7% in the study by McDowell et al. (10) and 3.0% in the study by Huang et al. (31), respectively. By contrast, only one patient (0.4%) developed grade 3 neck fibrosis as of the last follow-up in our study. Also, Sommat et al. reported that the 2-year incidence rate of hypothyroidism for patients receiving bilateral whole neck irradiation was 44.5% (32) whereas 38.6% of patients developed hypothyroidism as of the last follow-up in our study. This might be attributed to the fact that omitting the irradiation of the contralateral lower neck could decrease the exposure dose to the neighboring normal organs and tissues, including cervical subcutaneous tissues and thyroids. Although we lacked a control group of whole neck irradiation, our data showed that contralateral lower neck sparing radiotherapy might have the potential to improve the long-term quality of life of patients.

Therefore, based on these findings, it can be considered that omitting elective neck irradiation to the contralateral lower neck for patients with stage N1 NPC was safe and feasible.

There are several limitations of this study that need to be noted. First, since the present study was retrospective, the results might have been affected by bias in the data collection. Second, we lacked a control group in which patients received whole neck irradiation. Third, the chemotherapy regimens used were not completely identical. We expect that large-scale randomized controlled clinical trials will be conducted in the near future to address these issues.

## Conclusion

According to our study, the incidence of out-of-field lymph node recurrence was rare when elective neck irradiation of the contralateral lower neck was omitted in patients with stage N1 NPC. Contralateral lower neck sparing radiotherapy would be safe and feasible for patients with stage N1 NPC, with the potential to improve the long-term quality of life of patients.

## Data Availability Statement

The raw data supporting the conclusions of this article will be made available by the authors, without undue reservation.

## Ethics Statement

The studies involving human participants were reviewed and approved by the ethics committee of Sun Yat-sen University Cancer Center. Written informed consent for participation was not required for this study in accordance with the national legislation and the institutional requirements. Written informed consent was obtained from the individual(s) for the publication of any potentially identifiable images or data included in this article.

## Author Contributions

Study design: CZ, HW, and FH. Data collection: ZS, JW, RH, and XW. Quality control of data: CC and MD. Data analysis and interpretation: ZS, JW, and RH. Manuscript preparation: ZS, JW, and RH. Manuscript editing: HW and FH. All authors contributed to the article and approved the submitted version.

## Conflict of Interest

The authors declare that the research was conducted in the absence of any commercial or financial relationships that could be construed as a potential conflict of interest.
